# Identifying Mitigation Pathways for Low‐Carbon Strawberry Production under Mediterranean Conditions: A Life Cycle Assessment

**DOI:** 10.1002/gch2.70120

**Published:** 2026-06-08

**Authors:** Ege Balkiş, Olcay Gülçiçek Uysal

**Affiliations:** ^1^ Faculty of Engineering Department of Environmental Engineering Mersin University Mersin Türkiye

**Keywords:** carbon footprint, life cycle assessment, mediterranean agriculture, mitigation pathways, open‐field strawberry, sensitivity analysis, yield sensitivity

## Abstract

Agricultural production is a significant contributor to global greenhouse gas emissions, highlighting the need for region‐specific assessments of climate impacts across food systems. This study evaluates the carbon footprint of open‐field strawberry production in the Silifke district (Mersin, Türkiye) using a process‐based Life Cycle Assessment approach with a cradle‐to‐farm‐gate system boundary and a functional unit of 1 kg of strawberries. Life cycle modeling was performed in SimaPro using background data from Ecoinvent 3.10, and impacts were quantified using the IPCC 2021 Global Warming Potential method. The results indicate a total carbon footprint of 0.25052 kg CO_2_‐eq kg^−^^1^, placing the system within the lower range reported for Mediterranean strawberry production. The cultivation stage was the dominant contributor (77.56%), followed by raw material supply (22.44%). Fertilizer use, particularly nitrogen, phosphorus, and potassium (NPK) fertilizers, was identified as the primary emission hotspot. Scenario analysis showed that a 20% reduction in fertilizer application decreases the carbon footprint by 9.83%, while solar‐powered irrigation results in only a minor reduction (0.56%). Sensitivity analysis confirmed fertilizer inputs as the dominant emission driver (S ≈ 0.49), while electricity consumption has negligible influence. Yield sensitivity further showed that reduced productivity significantly increases carbon footprint, highlighting fertilizer optimization and yield stability as key mitigation strategies.

## Graphical abstract

This study evaluates the carbon footprint of open‐field strawberry production under Mediterranean conditions using a life cycle assessment approach. Fertilizer use, particularly nitrogen, phosphorus, and potassium (NPK), is identified as the dominant emission source. Results show that improving fertilizer efficiency and maintaining yield stability are the most effective strategies for reducing emissions and enhancing environmental sustainability.

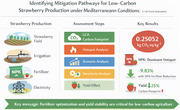

## 1 Introduction

Agricultural production is a major contributor to global greenhouse gas (GHG) emissions, accounting for approximately 10%–12% of total anthropogenic emissions when agriculture, forestry, and other land use activities are considered. As a result, the accurate quantification of environmental impacts associated with food production systems has become a critical component of climate change mitigation strategies. LCA has therefore emerged as a widely accepted methodological framework for evaluating the environmental performance of agricultural products across different production stages and management practices [1].

Recent systematic reviews and meta analyses have shown that fruit and vegetable production systems generally exhibit lower carbon footprints compared to animal based food products, while still displaying substantial variability depending on methodological choices, system boundaries, and regional conditions. In a comprehensive meta‐analysis covering 118 LCA studies, Mandouri et al. [2] demonstrated that fruits and vegetables consistently show lower greenhouse gas emission intensities, although significant differences arise due to input intensity, energy use, and production system characteristics. These findings highlight the importance of crop and region specific assessments to accurately identify emission drivers and mitigation opportunities.

The Mediterranean region is widely recognized as one of the global hotspots of climate change, characterized by increasing temperatures, altered precipitation regimes, and growing water scarcity. Agricultural systems in this region are particularly sensitive to climate related pressures due to intensive input use, especially mineral fertilizers, fossil fuel based mechanization, and irrigation related energy consumption. Consequently, Mediterranean agroecosystems contribute substantially to greenhouse gas emissions while simultaneously facing constraints related to water availability and soil degradation [3, 4]. These vulnerabilities underscore the need for system specific LCA studies capable of capturing the interactions between climate conditions, management practices, and environmental impacts [5].

Recent European and global assessments have emphasized that the carbon footprint of strawberry production varies widely across cultivation systems. Open‐field strawberry production systems generally exhibit relatively low climate change impacts, whereas greenhouse and highly controlled production systems are often dominated by infrastructure and energy related emissions, particularly heating and electricity demand [6, 7]. Similar patterns have been reported for intensive indoor and controlled environment strawberry production systems, where continuous cooling, lighting, and ventilation requirements substantially increase energy demand and associated carbon emissions [8]. These findings indicate that production system characteristics, rather than crop type alone, play a decisive role in shaping the environmental performance of strawberry cultivation.

Within this context, the Mediterranean region represents a critical case for evaluating the sustainability of strawberry production. Mediterranean agroecosystems are inherently constrained by limited water availability, high evapotranspiration rates, and relatively low soil carbon sequestration potential, which together amplify the environmental consequences of intensive agricultural practices [9]. Recent studies further emphasize that Mediterranean agriculture is increasingly challenged by the combined pressures of greenhouse gas emissions, water scarcity, and rising energy demand, highlighting the importance of life cycle based assessments for identifying environmentally efficient cropping systems [10].

Despite the growing body of LCA literature on agricultural systems, studies focusing on Mediterranean agriculture based on site specific, farm level data remain limited. Methodological challenges related to spatial and temporal variability, as well as data availability, continue to constrain the accuracy of agricultural GHG emission estimates. Recent research has highlighted that integrating detailed field data into LCA frameworks can substantially improve the reliability and policy relevance of environmental impact assessments [1].

Strawberry production in Türkiye provides a particularly relevant case for such assessments. Reviews focusing on the water–energy–food–ecosystem (WEFE) nexus emphasize the importance of climate smart and resource efficient farming practices in Mediterranean agriculture, especially for high value horticultural crops [11]. At the global scale, fruit and vegetable production systems are increasingly recognized as key contributors to climate change mitigation due to their relatively low carbon footprints compared to animal based foods, while still requiring targeted interventions to further reduce emissions under climate vulnerable conditions [12]. Strawberry production represents a strategically important horticultural activity in Türkiye, both in terms of domestic supply and export oriented fresh fruit markets. Owing to its favorable Mediterranean climate, extended growing season, and well developed horticultural infrastructure, Türkiye ranks among the leading strawberry producing countries in the region. Within this national context, Mersin Province plays a central role, with the Silifke district emerging as the dominant production hub. According to the Silifke Chamber of Commerce and Industry [13], a substantial share of Türkiye's strawberry production (around 40%) and a dominant share of national strawberry exports (approximately 60%), highlighting the district's exceptional contribution to national and international markets. Within this broader policy and technology framework, farm level carbon footprint assessments of specific crops and production systems are essential to support evidence based sustainability transitions. Recent policy oriented assessments emphasize that export oriented agrifood systems increasingly require crop specific and regionally representative carbon footprint evidence to remain competitive under evolving sustainability criteria in international markets [11, 12]. In this context, farm level life cycle assessments provide a critical decision support tool for identifying low carbon production pathways in climate vulnerable regions such as the Mediterranean.

Beyond production volume, the strategic importance of Silifke strawberry production is further reinforced by its geographical indication (GI) status, officially granted in 2019 [14]. The GI certification reflects distinctive quality attributes linked to local agroclimatic conditions and enhances market visibility and export competitiveness. As documented in regional development and sectoral reports, Silifke strawberries are widely exported to international markets, particularly to the Russian Federation and European countries, positioning the region as a key node in Türkiye's fresh fruit supply chains [13, 15].

In this context, assessing the carbon footprint of open field strawberry production in Silifke is of particular relevance. Given the growing emphasis on environmental performance, sustainability labeling, and climate related trade requirements in agri food markets, region specific life cycle assessments provide critical evidence for evaluating both environmental efficiency and long term competitiveness of geographically indicated agricultural products. Beyond its regional significance, the Silifke case provides a globally representative example of open field, export oriented strawberry production under Mediterranean climatic conditions.

Therefore, this research addresses a significant gap in the literature by delivering a detailed, site specific assessment of greenhouse gas emissions based entirely on farm level primary data derived from a GI certified Mediterranean production system. The findings offer transferable and policy relevant insights for climate vulnerable regions seeking to reconcile export competitiveness with low carbon agricultural production. Accordingly, the primary objective of this study is to quantify the carbon footprint of Silifke strawberry production using a process based LCA framework and to identify key emission hotspots, thereby providing evidence based support for targeted mitigation strategies and sustainability oriented agricultural policies.

## 2 Materials and Methods

### 2.1 Scope and Methodological Framework

In this study, the carbon footprint of open‐field strawberry production in the Silifke district of Mersin Province, Türkiye, was assessed with a focus on farm‐level greenhouse gas emissions. The analysis covered all production stages of strawberry cultivation, ranging from land preparation to harvest. Environmental impacts were evaluated using the LCA approach. The LCA was conducted in accordance with ISO 14040 and ISO 14044 standards. Modeling and calculations were performed using SimaPro 10.1.0.6 software, with background data obtained from the Ecoinvent 3.10 database. The definition of system boundaries was informed by relevant guidance provided under the Product Environmental Footprint Category Rules [16] for fruits and vegetables.

#### 2.1.1 Study Area

**FIGURE 1:**
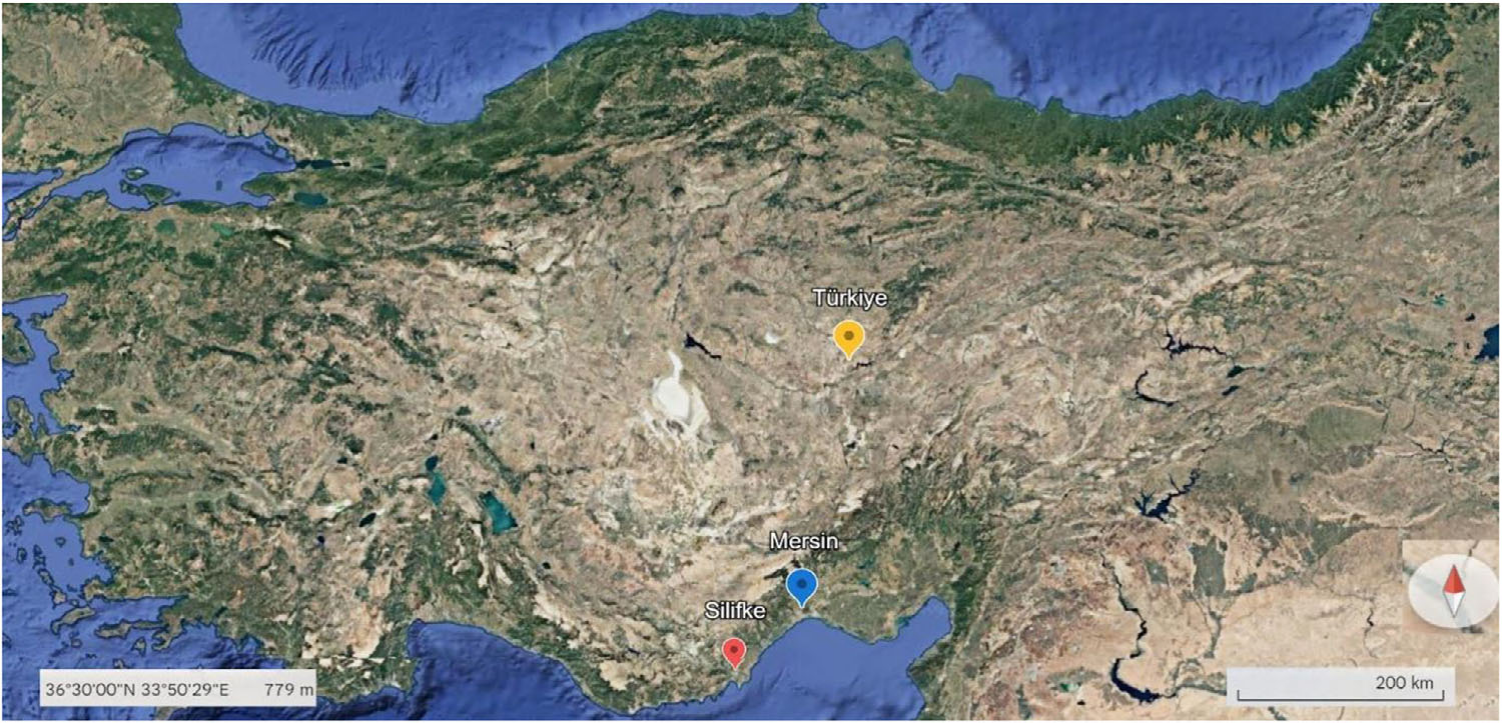
Geographical location of the study area: Mersin province and Silifke district in the Mediterranean region of Türkiye.

The study was conducted in the Silifke district of Mersin Province, located in the Mediterranean Region of Türkiye (Figure 1). Silifke is one of the major strawberry producing areas in the country due to its favorable climatic conditions, fertile alluvial soils, and well developed irrigation infrastructure. The district is situated close to the Mediterranean coast at an elevation of approximately 5–10 m above sea level and is characterized by a typical Mediterranean climate, with mild and rainy winters and hot, dry summers.

Strawberry production in the region is predominantly carried out under open‐field conditions, supported by drip irrigation systems. According to regional agricultural statistics, Silifke accounts for a substantial share of strawberry production in Mersin Province, which is among the leading strawberry producing provinces in Türkiye. These characteristics make Silifke a representative and relevant study area for assessing the carbon footprint of open‐field strawberry production.

### 2.2 Life Cycle Assessment Framework

#### 2.2.1 System Boundaries and Functional Unit

The LCA was conducted using a cradle‐to‐farm gate system boundary. Accordingly, the system included all processes from land preparation and input production to the harvested strawberries leaving the farm gate. Processes related to post harvest storage, packaging, distribution, retail, and consumption were excluded from the system boundaries.

Recent studies have emphasized the importance of identifying emission hotspots at the farm level using life cycle‐based approaches in agricultural systems [3]. In line with this approach, a cradle‐to‐farm gate LCA framework was adopted in the present study to quantify the carbon footprint of open‐field strawberry production under Mediterranean conditions. This system boundary was deliberately selected because on‐farm processes are typically the dominant contributors to greenhouse gas emissions in agricultural production systems.

Extending the system boundary beyond the farm gate (e.g., transportation, packaging, distribution, and consumption) would introduce additional variability and uncertainty due to region‐specific supply chain structures, logistics, and consumer behavior. Therefore, limiting the system boundary to the farm gate ensures methodological consistency, reduces uncertainty, and allows for better comparability with similar agricultural LCA studies conducted under Mediterranean conditions. The functional unit (FU) was defined as the production of 1 kg of fresh strawberries at the farm gate, which allows for a consistent comparison of environmental impacts across production inputs and processes.

Previous LCA studies on strawberry production have adopted broader system boundaries extending beyond the farm gate. For example, Parajuli et al. [17] conducted a cradle‐to‐grave LCA of open‐field strawberry production in California, incorporating post farm stages such as packaging, cooling, transportation, retail, consumer use, and food waste management. The substantially higher carbon footprint reported in that study reflects the inclusion of downstream supply chain processes rather than differences in on farm production practices. This comparison highlights the influence of system boundary selection on carbon footprint results.

#### 2.2.2 Data Sources and Inventory Development

Data used for the life cycle inventory (LCI) were obtained from both primary and secondary sources. Primary data were collected between 2023 and 2025 through field surveys and direct interviews with commercial strawberry producers operating in the Silifke district, as well as from records of the Silifke Chamber of Agriculture and information provided by the State Hydraulic Works (DSİ) and local irrigation associations. Secondary data were obtained from the Turkish Statistical Institute and relevant scientific literature [18]. The use of primary, farm specific data is widely recommended in agricultural LCA studies to improve the representativeness and reliability of inventory results, particularly under region‐specific production conditions [3].

According to the 2025 records of the Silifke Chamber of Agriculture, strawberry production in the study area is carried out on a total area of 315 ha, with an average planting density of 55 000 seedlings per hectare [13]. Information obtained from producer interviews includes data on the types, application rates, and frequencies of fertilizers and pesticides used in the study area. Fertilizer application consists mainly of compound NPK fertilizers, potassium, phosphorus, and ammonium‐based inputs applied throughout the growing season. Plant protection practices primarily involve fungicide applications under routine management conditions. In addition, crop management inputs expressed on a per hectare basis are presented in Table 1.

**TABLE 1:** Crop management inputs for open‐field strawberry production in Silifke (per hectare basis).

Input category	Input type	Amount	Unit	Description
Planting	Seedlings	55 000	units/ha	Average planting density
Fertilizers	NPK fertilizers	400	kg/ha	20 applications × 20 kg/ha
Fertilizers	Potassium fertilizer	200	kg/ha	10 applications × 20 kg/ha
Fertilizers	Phosphorus fertilizer	20	kg/ha	Single application
Fertilizers	Ammonium sulfate	200	kg/ha	10 applications × 20 kg/ha
Fertilizers	Rooting fertilizer	4	kg/ha	2 applications × 2 kg/ha
Plant protection	Fungicide	10	kg/ha	20 applications × 0.5 kg/ha
Irrigation	Water use	7500–10 000	m^3^/ha	Total during cropping cycle
Energy	Electricity consumption	∼30 600	kWh/ha	170 kWh/day × ∼180 days
Machinery	Diesel (field operations)	125	L/ha	Soil preparation & cultivation
Machinery	Diesel (harvest transport)	960	L/ha	Labor transport during harvest
Yield	Strawberry production	40 000	kg/ha	Average yield

*Note*: All values represent average field conditions based on producer surveys and regional data sources. Irrigation water refers to the total amount applied during the cropping cycle (from transplanting to harvest).

Fuel consumption associated with land preparation and soil cultivation activities carried out using tractors and similar agricultural machinery was estimated at an average of 125 L/ha. During the harvest period, which lasts approximately three months, diesel fuel consumption associated with labor transportation to the fields averages 80 L per week, resulting in a total consumption of approximately 960 L/ha over the harvest season.

Irrigation in strawberry production is performed using drip irrigation systems. Data obtained from the State Hydraulic Works (DSİ) and local irrigation associations indicate that approximately 7,500–10,000 m^3^/ha of irrigation water is applied annually. These values are based on official regional records and are consistent with field observations obtained from local producers. The reported irrigation volumes represent the total water applied during the cropping cycle (from transplanting to harvest). The energy required to operate the irrigation systems is supplied exclusively by electricity. According to producer information, the average daily electricity consumption is approximately 170 kWh, which was aggregated over an approximately six‐month growing period to estimate total electricity use per hectare. The relatively high irrigation demand compared to values reported in other Mediterranean regions can be attributed to local climatic conditions, soil characteristics, and irrigation management practices specific to the study area. This higher irrigation demand may also be associated with high evapotranspiration rates under Mediterranean climatic conditions and the need for frequent irrigation in well‐drained soils to maintain stable soil moisture during intensive production periods.

The functional unit of the study was defined as 1 kg of fresh strawberries produced in Silifke. However, since field data were generally recorded as total input amounts per decare (e.g., fertilizers, pesticides, water, and fuel), all inventory data were converted to the functional unit for implementation in the SimaPro software. Based on field data indicating that 55000 seedlings are planted per hectare and an average yield of 40,000 kg strawberries /ha, it was calculated that 1.375 seedlings are required to produce 1 kg of strawberries. All production inputs were normalized accordingly to represent the functional unit. This normalization approach is consistent with standard practice in crop based LCA studies, where field level input data are converted to a mass‐based functional unit to enable comparability across production systems [19].

The resulting inventory data for 1 kg of Silifke strawberries are summarized in Table 2. The developed inventory represents the open‐field strawberry production system currently practiced in the Silifke region and constitutes the basis for the carbon footprint assessment.

**TABLE 2:** Inputs required for the production of 1 kg of Silifke strawberries.

Input	Amount required for 1 kg strawberry production	Unit
Number of seedlings	1.375	Unit
Seedling supplier location	Nevşehir city	—
Transportation distance	351	Km
Transportation vehicle and fuel type	Light‐duty truck (diesel)	—
Growing medium type	Peat	—
Growing medium amount	50	G
Irrigation water amount	0.200000	m^3^
Electricity consumption	0.002420	kWh
Ammonium sulfate	0.005000	Kg
NPK fertilizers	0.010000	Kg
Rooting fertilizer	0.000100	Kg
Phosphorus fertilizer	0.000500	Kg
Potassium fertilizer	0.005000	Kg
Fungicide	0.000250	Kg
Diesel consumption for tractor plowing	0.000625	L
Diesel consumption for subsoiler use	0.000625	L
Diesel consumption for rotary tiller	0.000625	L
**Diesel consumption for bed formation operation**	0.000250	L
**Diesel consumption for plastic mulch laying**	0.000250	L
Diesel consumption for labor transportation during harvesting	0.000076	L

*Note*: All values expressed in liters (L) for machinery operations represent diesel fuel consumption associated with the respective agricultural activities.

#### 2.2.3 Impact Assessment Method and Carbon Footprint Calculation

The carbon footprint of strawberry production was assessed using a life cycle assessment (LCA) framework with a primary focus on climate change impacts. Greenhouse gas emissions associated with the production system were quantified using the Global Warming Potential over a 100‐year time horizon (GWP100), based on characterization factors from the IPCC Sixth Assessment Report [20]. All emissions were expressed as kilograms of carbon dioxide equivalents (kg CO_2_‐eq) per functional unit, defined as 1 kg of fresh strawberries at the farm gate.

Carbon footprint calculations were performed using the life cycle inventory developed in this study and implemented in SimaPro 10.1.0.6 software, with background datasets sourced from the Ecoinvent 3.10 database. This modelling approach enables a transparent, reproducible, and internationally comparable assessment of greenhouse gas emissions associated with open‐field strawberry production in the Silifke region. The application of IPCC‐based characterization factors is widely adopted in agricultural LCA studies and ensures consistency with international climate reporting frameworks.

Processes related to the manufacture of agricultural machinery, infrastructure, capital goods, and inputs used in negligible quantities were excluded from the system boundaries, as their contribution to total greenhouse gas emissions in farm‐level agricultural systems is generally limited. This approach is consistent with common practice in agricultural LCAs, where emphasis is placed on operational inputs and direct emission sources that dominate climate change impacts.

### 2.3 Impact Categories

The selection of climate change as the primary impact category in this study is consistent with recent LCA literature, which identifies Global Warming Potential (GWP) as the most widely applied and policy‐relevant indicator in food production systems [2]. Carbon footprint indicators are particularly suitable for evaluating agricultural production systems due to their direct relevance to climate mitigation strategies and greenhouse gas reduction targets.

In Mediterranean agricultural contexts, comprehensive LCA studies often consider multiple environmental impact categories, including climate change, acidification, eutrophication, photochemical oxidation, and resource use [5]. However, the present study focuses exclusively on climate change impacts in order to provide a targeted assessment of greenhouse gas emissions associated with open‐field strawberry production.

The focus on climate change impacts using the [20] GWP100 methodology is consistent with recent LCA studies on Mediterranean agricultural systems that prioritize carbon footprint indicators for farm level emission assessments [1]. Although multi impact assessments can offer a broader environmental perspective, concentrating on a single, well established indicator allows for clearer interpretation and improved comparability of results across studies.

While comprehensive agri environmental assessments may include additional impact categories such as acidification, eutrophication, ozone depletion, and cumulative energy demand [10], the present study deliberately limits its scope to climate change impacts (GWP100) to deliver a focused and policy relevant evaluation of greenhouse gas emissions under Mediterranean open‐field production conditions.

In this context, while the study focuses on climate change impacts using the GWP100 indicator, it is acknowledged that agricultural sustainability involves multiple environmental dimensions. Therefore, while carbon footprint provides a robust and policy‐relevant metric for assessing greenhouse gas emissions, it should be interpreted as one component of a broader environmental assessment framework. This perspective ensures that the results are interpreted within a wider sustainability context, while maintaining a focused and comparable assessment approach.

### 2.4 Scenario and Sensitivity Analysis

To evaluate the mitigation potential and robustness of the calculated carbon footprint, scenario and sensitivity analyses were conducted based on the identified hotspot processes.

#### 2.4.1 Scenario Analysis

Four scenarios were defined to assess the mitigation potential of key emission sources identified in the hotspot analysis. Scenario 1 represents the baseline system reflecting current farming practices in the study area. Scenario 2 evaluates the effect of reduced fertilizer application by decreasing NPK inputs by 20% relative to baseline conditions. This reduction range is supported by previous studies indicating that moderate reductions in nitrogen inputs (10%–20%) can be achieved without significant yield loss while reducing greenhouse gas emissions [21]. In addition, European policy frameworks commonly adopt a 10%–20% fertilizer reduction range as a realistic target for sustainable agricultural systems [22], and fertilizer optimization has been identified as a key mitigation strategy in Mediterranean agricultural conditions [23].

Scenario 3 evaluates energy‐related mitigation by replacing grid electricity with renewable energy sources (solar‐powered irrigation), thereby eliminating electricity‐related emissions at the operational level. In this scenario, electricity‐related emissions were assumed to be reduced to zero (i.e., −100% change in electricity consumption). The feasibility of solar energy adoption is supported by the high solar radiation and long sunshine duration observed in Türkiye, particularly in the Mediterranean region and Mersin province [24, 25]. Local‐scale studies further confirm the high solar energy potential of Mersin based on long‐term observations [26].

Scenario 4 combines both strategies, integrating a 20% reduction in NPK fertilizer use and the complete substitution of grid electricity with renewable energy. These scenarios were designed to identify the relative contribution of different mitigation pathways and to quantify their effects on the overall carbon footprint.

#### 2.4.2 Input‐Based Sensitivity Analysis

A deterministic one‐at‐a‐time (OAT) sensitivity analysis was conducted to quantify the influence of key input parameters on the carbon footprint. Based on the hotspot analysis, total NPK fertilizer application (expressed as the combined mass of nitrogen, phosphorus, and potassium fertilizers applied per hectare) and electricity consumption for irrigation were identified as the most influential parameters. Electricity consumption in this context refers specifically to the energy required for the operation of drip irrigation systems. Each parameter was varied independently by ±20% relative to baseline conditions while keeping all other variables constant, following a deterministic OAT approach. In addition, a separate scenario analysis was conducted for electricity consumption, including a full reduction case (−100%), to represent a potential transition to renewable energy use. The sensitivity coefficient (*S*) was calculated as:

$$S=\frac{\Delta CF\left(\%\right)}{\Delta Input\left(\%\right)}$$

where Δ*CF* represents the percentage change in carbon footprint and Δ*Input* denotes the percentage change in the corresponding input parameter.

#### 2.4.3 Yield Sensitivity Analysis

A yield‐based sensitivity analysis was performed to evaluate the influence of crop productivity on the carbon footprint. Since the carbon footprint is expressed per unit of product, variations in yield directly affect the results. The carbon footprint (CF) is defined as:

$$CF=\frac{E}{Y}$$

where *E* represents total emissions and *Y* denotes crop yield. In this analysis, yield was varied by ±20% while total emissions were kept constant, and the resulting changes in CF were calculated based on the inverse relationship between yield and carbon footprint. All calculations were performed using a percentage‐based variation approach implemented in Microsoft Excel.

## 3 Results and Discussion

### 3.1 Carbon Footprint of Open‐Field Strawberry Production

The carbon footprint of open‐field strawberry production in the Silifke district was quantified using the LCA framework with a cradle‐to‐farm gate system boundary and a functional unit of 1 kg of fresh strawberries. Calculations were performed using SimaPro 10.1.0.6 software with background data from the Ecoinvent 3.10 database.

The results indicate that the total carbon footprint associated with the production of 1 kg of Silifke strawberries is 0.25052 kg CO_2_‐eq kg^−^^1^ product. This value represents the cumulative greenhouse gas emissions arising from all processes included within the system boundaries, encompassing raw material supply, cultivation activities, fertilizer and pesticide use, irrigation related energy consumption, agricultural machinery operation, and on farm transportation.

When compared with values reported in the literature, the estimated carbon footprint of Silifke strawberries can be considered relatively low. Recent LCA studies have reported a wide range of carbon footprint values for fruit and vegetable production systems, reflecting differences in crop type, input intensity, system boundaries, and methodological choices [2, 5]. Accordingly, the value obtained in this study places Silifke strawberry production within the group of fruit products characterized by a comparatively low climate change impact.

The relatively low carbon footprint observed in this study can be primarily attributed to the open‐field production system applied in the Silifke region, which does not require energy intensive infrastructure such as greenhouse structures or climate control systems. This interpretation is consistent with findings reported for Mediterranean cropping systems, where fertilization, fuel consumption, and irrigation related processes dominate greenhouse gas emissions, while infrastructure‐related inputs constitute major emission hotspots in protected cultivation systems [5, 10].

Similar patterns have been reported in strawberry specific studies. Frakolaki et al. [27] demonstrated that open‐field strawberry cultivation in Southeastern Europe systematically results in lower carbon footprint values compared to protected and soilless systems, primarily due to the absence of plastic tunnels, substrates, and permanent infrastructure. Likewise, Galafton et al. [28] showed that strawberry production systems without permanent protective structures tend to exhibit lower climate change impacts, whereas greenhouse and macro‐tunnel systems are dominated by infrastructure‐related emissions.

Overall, these findings demonstrate that open‐field strawberry production in Silifke exhibits a relatively low carbon footprint under current management practices and falls well within the range reported for Mediterranean agricultural systems.

### 3.2 Contribution of Life Cycle Stages to the Carbon Footprint

The contribution of different life cycle stages to the total carbon footprint of open‐field strawberry production in Silifke was evaluated by grouping the production system into two main stages: raw material supply and cultivation. This stage‐based approach allows the identification of major emission drivers along the production chain and supports the interpretation of mitigation priorities at farm level.

**FIGURE 2:**
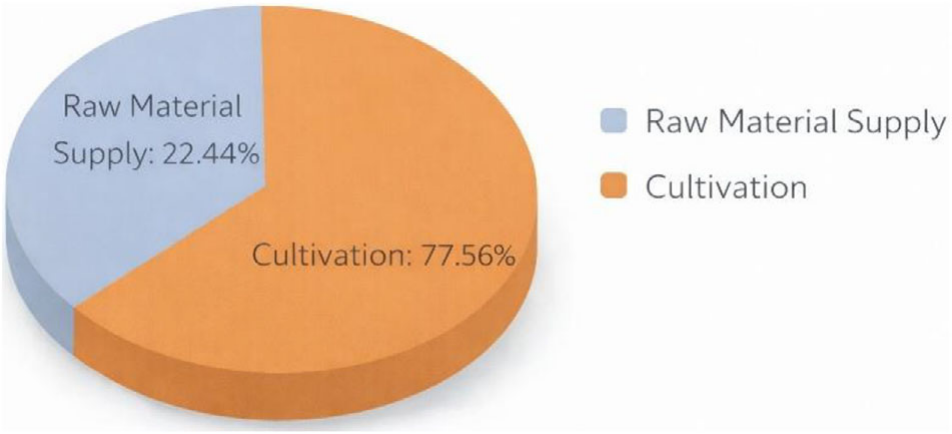
Contribution of raw material supply and cultivation stages to the climate change potential (GWP100) of open‐field strawberry production in Silifke, Türkiye.

The relative contributions of the raw material supply and cultivation stages to the climate change potential (GWP100) are illustrated in Figure 2, while a detailed breakdown of environmental impact categories and their distribution between life cycle stages is presented in Table 3.

**TABLE 3:** Environmental impact results of open‐field strawberry production in Silifke, Türkiye.

Impact category	Unit	Total	Raw material supply	Cultivation
Climate change	kg CO_2_ eq	0.25052	0.05642	0.19416
Ozone depletion	kg CFC‐11 eq	6.6 × 10^−^^9^	8.1 × 10^−^^1^ ^1^	6.5 × 10^−^^9^
Ionizing radiation	kBq U‐235 eq	0.01488	0.00036	0.01451
Photochemical ozone formation	kg NMVOC eq	0.00087	0.00003	0.00084
Particulate matter	disease inc.	1.5 × 10^−^^8^	3.9 × 10^−^^10^	1.5 × 10^−^^8^
Human toxicity, non‐cancer	CTUh	1.1 × 10^−^^7^	6.6 × 10^−^^1^ ^1^	1.1 × 10^−^^7^
Human toxicity, cancer	CTUh	1.3 × 10^−^^9^	2.16 × 10^−^^1^ ^1^	1.3 × 10^−^^9^
Acidification	mol H^+^ eq	0.002	2.81 × 10^−^^5^	0.00197
Freshwater eutrophication	kg P eq	0.00012	1.61 × 10^−^^6^	1.2 × 10^−^^4^
Marine eutrophication	kg N eq	0.00137	7.93 × 10^−^^6^	1.3 × 10^−^^3^
Terrestrial eutrophication	mol N eq	0.00634	8.4 × 10^−^^5^	0.00626
Freshwater ecotoxicity	CTUe	995.2187	0.034922	995.18377
Land use	Pt	2.91940	2.14574	0.77366
Water use	m^3^ depr.	14.71346	0.38177	14.3316
Resource use, fossils	MJ	1.6373	0.63603	1.00130
Resource use, minerals and metals	kg Sb eq	0.00001	3.2 × 10^−^^8^	1.3 × 10^−^^5^
Climate change, fossil	kg CO_2_ eq	0.250204	0.056415056	0.19378
Climate change, biogenic	kg CO_2_ eq	0.00019	3.3 × 10^−^^6^	0.00018
Climate change, land use and land use change	kg CO_2_ eq	0.00019	6.3 × 10^−^^6^	0.00018
Human toxicity, non‐cancer (metals)	CTUh	4.2 × 10^−^^9^	5.1 × 10^−^^1^ ^1^	4.2 × 10^−^^9^
Human toxicity, cancer (organic)	CTUh	1.3 × 10^−^^9^	2.1 × 10^−^^1^ ^1^	1.3 × 10^−^^9^
Human toxicity, cancer (inorganic)	CTUh	0	0	0
Human toxicity, cancer (metals)	CTUh	3.8 × 10^−^^1^ ^1^	5.8 × 10^−^^1^ ^3^	3.8 × 10^−^^1^ ^1^
Freshwater ecotoxicity (organics)	CTUe	991.24375	0.00627	991.23748
Freshwater ecotoxicity (inorganics)	CTUe	2.54604	0.00544	2.54059
Freshwater ecotoxicity (metals)	CTUe	1.42890	0.02320	1.40569

Beyond climate change impacts, Table 3 indicates that the cultivation stage also dominates several additional midpoint impact categories, including freshwater eutrophication, marine eutrophication, acidification, and freshwater ecotoxicity. These impacts are primarily associated with fertilizer application and on farm input use, highlighting that cultivation stage practices exert multidimensional environmental pressures beyond greenhouse gas emissions. In contrast, the raw material supply stage shows a relatively higher contribution to land use impacts, reflecting the role of upstream nursery production and growing media use. This distribution confirms that mitigation strategies targeting cultivation stage inputs can simultaneously deliver co benefits across multiple environmental impact categories.

In particular, the dominance of eutrophication related impacts highlights the close link between nutrient management and broader environmental performance in Mediterranean agricultural systems. Similar patterns have been reported in previous life cycle based assessments, where fertilizer use simultaneously drives climate change, eutrophication, and acidification impacts [3, 5]. This finding reinforces the importance of integrated mitigation strategies that address multiple environmental objectives rather than focusing solely on carbon footprint reduction.

The results indicate that the cultivation stage is the dominant contributor, accounting for 77.56% of the total carbon footprint of open‐field strawberry production. This stage includes on farm activities such as fertilizer and pesticide application, irrigation related electricity consumption, agricultural machinery use, soil preparation operations, and labor transportation during the harvest period. The high contribution of the cultivation stage reflects the intensity of input use and energy demand associated with crop management practices under open‐field conditions.

In contrast, the raw material supply stage contributes 22.44% of the total carbon footprint. This stage primarily comprises emissions associated with the production and transportation of seedlings, growing media, fertilizers, and other agricultural inputs prior to their use at the farm level. Within this stage, seedling production and long distance transportation represent the main emission sources, owing to the energy and material requirements of nursery production and logistics.

A similar dominance of the cultivation stage over upstream input supply has been widely reported for Mediterranean agricultural systems assessed using life cycle based approaches, where on farm management practices typically outweigh raw material supply in terms of climate change impacts [3, 5].

Overall, the dominance of the cultivation stage highlights the critical role of on farm management practices in shaping the carbon footprint of open‐field strawberry production in Silifke. These findings indicate that mitigation strategies aiming to reduce greenhouse gas emissions should primarily focus on optimizing cultivation related inputs and processes, particularly fertilizer management, energy use for irrigation, and fuel consumption associated with agricultural machinery. Improvements in input sourcing and logistics within the raw material supply stage may provide additional, though secondary, opportunities for emission reduction.

Although climate change potential (GWP100) constitutes the primary focus of this study, other environmental impact categories also provide important insights into the sustainability profile of Silifke strawberry production. Among these, water use emerges as a particularly relevant impact category, with a total freshwater consumption of 14.71 m^3^ depriv. per kg of strawberry. This result reflects the irrigation intensive nature of strawberry cultivation under Mediterranean climatic conditions and is consistent with the regional dependence on supplemental irrigation, especially considering the increasing water scarcity risks in the Mediterranean basin.

In addition, freshwater and marine eutrophication impacts are mainly driven by fertilizer application during the cultivation stage, indicating the potential risk of nutrient losses to surrounding water bodies. These findings underline the importance of integrated nutrient and water management strategies, not only to reduce greenhouse gas emissions but also to minimize pressure on local water resources and aquatic ecosystems.

#### 3.2.1 Raw Material Supply Stage

Greenhouse gas emissions associated with the raw material supply stage are primarily driven by strawberry seedling production. This process includes the use of peat‐based growing media, energy consumption during nursery operations, and post production handling activities prior to delivery to the farm. According to the LCA results, strawberry seedling production resulted in 0.05622 kg CO_2_‐eq, accounting for 22.44% of the total carbon footprint and representing almost the entire contribution of the raw material supply stage (Table 4).

**TABLE 4:** Percentage contribution of production inputs to the total carbon footprint of strawberry production within the system boundaries.

Input	kg CO_2_‐eq	Contribution (%)
Strawberry seedlings	0.05622	22.44
Irrigation water use	0.01705	6.81
Electricity consumption	0.00140	0.56
Ammonium sulfate	0.00927	3.70
NPK fertilizers	0.12322	49.17
Rooting fertilizer	0.00116	0.46
Phosphorus fertilizer	0.00089	0.36
Potassium fertilizer	0.00178	0.71
Fungicide	0.00049	0.20
Use of agricultural machinery	0.01597	6.38
Transportation of labor for harvesting	0.00028	0.12
Transportation of raw materials	0.00019	0.08
Strawberry production (open‐field level direct emissions)	0.02260	9.02
TOTAL	0.25052	100.00

Similar patterns have been reported for Mediterranean agricultural systems, where upstream input production particularly nursery‐based seedling production and growing media use constitutes a significant share of greenhouse gas emissions within the raw material supply stage [3, 5]. These studies highlight that energy use during seedling propagation and the production of peat‐based substrates are among the dominant emission sources before on farm activities begin.

Logistical activities considered within this stage involve the transportation of strawberry seedlings from Nevşehir to the Silifke district. The transportation process was modeled using a light duty diesel truck, consistent with the assumptions defined in the life cycle inventory. The contribution of seedling transportation to the total carbon footprint was calculated as 0.00019 kg CO_2_‐eq, corresponding to 0.08% of total emissions, indicating a relatively minor impact compared to emissions associated with seedling production itself.

Overall, these findings demonstrate that greenhouse gas emissions in the raw material supply stage are overwhelmingly dominated by upstream seedling production processes, while transportation related emissions remain negligible. This distribution is consistent with previous farm level LCA studies conducted under Mediterranean conditions, which emphasize that mitigation efforts targeting input supply chains should primarily focus on improving nursery production practices rather than short distance transportation logistics [3, 5]. Accordingly, strategies aimed at reducing the carbon footprint of open‐field strawberry production should prioritize growing media selection, energy efficiency in seedling production, and nursery management practices, while transportation related improvements are expected to yield only marginal emission reductions.

#### 3.2.2 Cultivation Stage

The cultivation stage represents the dominant contributor to the total carbon footprint of open‐field strawberry production in the Silifke region. As shown in Table 3, cultivation‐related activities account for 77.56% of the overall climate change impact (GWP100), corresponding to 0.19416 kg CO_2_‐eq per kg of strawberries. This percentage was calculated by dividing the cultivation‐stage emissions by the total carbon footprint (0.25052 kg CO_2_‐eq). Similar stage‐level dominance of on‐farm processes has been reported for Mediterranean agricultural systems assessed using life cycle‐based approaches, where cultivation activities typically outweigh upstream input supply in terms of climate change impacts [3, 5].

Within this stage, fertilizer application emerged as the primary hotspot. In particular, NPK fertilizers contributed 0.12322 kg CO_2_‐eq, representing 49.17% of the total carbon footprint (Table 4). The dominance of fertilizer related emissions is consistent with findings from Mediterranean and European agricultural LCAs, where fertilizer production and field level nitrogen‐related emissions repeatedly emerge as the leading drivers of climate change impacts [2, 4]. In strawberry specific studies conducted under Mediterranean conditions, fertilizer inputs have also been identified among the main contributors to on farm greenhouse gas emissions [19].

Irrigation related processes constituted the second most important contributor within the cultivation stage, accounting for 6.81% of total emissions (0.01705 kg CO_2_‐eq). This contribution aligns with Mediterranean crop LCAs reporting that irrigation influences carbon footprints mainly through energy demand for water pumping and the regional electricity mix rather than water application itself [1, 29].

The use of agricultural machinery, including soil preparation and field operations, contributed 6.38% (0.01597 kg CO_2_‐eq) to total emissions (Table 4). These impacts are primarily driven by diesel fuel combustion during tillage and mechanized field management activities, consistent with evidence from Mediterranean cropping systems where fuel related field operations constitute a relevant emission source [30, 31]. Electricity consumption associated with cultivation activities contributed only 0.56%, reflecting its relatively limited role compared to fuel based operations under the current system conditions.

Other inputs such as ammonium sulfate, potassium and phosphorus fertilizers, fungicides, and rooting agents individually contributed less than 4% each to the total carbon footprint. Labor transportation during the harvest period (i.e., transport of workers to the field as part of on‐farm harvesting operations within the system boundaries) showed a negligible contribution (0.12%), indicating that on‐farm production processes are far more influential than auxiliary logistical activities.

Overall, the results highlight that mitigation strategies aimed at reducing the carbon footprint of open‐field strawberry production should primarily focus on optimized fertilizer management, energy efficient irrigation practices, and reduced reliance on fossil fuel based machinery, as similarly emphasized across Mediterranean agricultural sustainability assessments [11, 12].

##### 3.2.2.1 Fertilizer Use as the Main Hotspot

Fertilizer use represents the dominant contributor to the carbon footprint of open‐field strawberry production in the Silifke district. As shown in Table 4, emissions associated with fertilizers account for a substantial share of total greenhouse gas emissions, with NPK fertilizers playing a particularly critical role.

The production and application of NPK fertilizers resulted in 0.12322 kg CO_2_‐eq, corresponding to 49.17% of the total carbon footprint. This high contribution is primarily attributed to the energy intensive manufacturing processes of synthetic fertilizers and to indirect emissions arising from nitrogen transformations in agricultural soils. In particular, post‐application nitrification and denitrification processes lead to the release of nitrous oxide (N_2_O), a greenhouse gas with a high global warming potential. Similar dominance of mineral fertilizers has been consistently reported in LCA studies of Mediterranean cropping systems, where nitrogen‐based fertilizers represent the main driver of climate change impacts [2, 4].

Ammonium sulfate contributed 0.00927 kg CO_2_‐eq, accounting for 3.70% of total emissions, whereas the contributions of phosphorus and potassium fertilizers remained relatively low, at 0.36% and 0.71%, respectively. These results highlight pronounced differences among fertilizer types in terms of their carbon footprint and emphasize that compound NPK fertilizers dominate overall fertilizer related emissions. Comparable patterns have been reported for open‐field strawberry production systems under Mediterranean conditions, where NPK fertilizers were identified as the primary emission hotspot, followed by nitrogen‐based supplementary fertilizers [19, 32].

The dominance of fertilizer related emissions observed in this study is consistent with broader evidence from Mediterranean agricultural systems, where nutrient management is recognized as a key factor in balancing productivity and climate mitigation objectives [9]. Recent meta‐analyses further confirm that across fruit and vegetable production systems, fertilizer use repeatedly emerges as the most influential contributor to greenhouse gas emissions, regardless of crop type or production intensity [2].

From a mitigation perspective, strategies aimed at reducing the carbon footprint of strawberry production in the Silifke region should therefore prioritize optimized fertilizer application rates, soil analysis based nutrient management, and improved nitrogen use efficiency. Such measures have been widely recommended as effective approaches for reducing greenhouse gas emissions in Mediterranean agricultural systems while maintaining crop productivity [11, 1].

##### 3.2.2.2 Irrigation and Electricity Consumption

Irrigation and electricity consumption constitute another important source of greenhouse gas emissions in open‐field strawberry production in the Silifke district. Strawberry cultivation in the study area relies predominantly on drip irrigation systems, which are widely adopted due to their high water use efficiency and suitability for Mediterranean climatic conditions.

According to the life cycle inventory results, irrigation water use contributed 0.01705 kg CO_2_‐eq, corresponding to 6.81% of the total carbon footprint (Table 4). Although drip irrigation is generally considered an efficient irrigation method in terms of water conservation, the associated environmental impact in this study is mainly driven by the energy demand required for water pumping rather than by water abstraction itself. Similar observations have been reported in Mediterranean agricultural systems, where irrigation related emissions are largely linked to indirect energy use instead of direct water consumption [29, 30].

Electricity consumption associated with irrigation system operation accounted for an additional 0.00140 kg CO_2_‐eq, representing 0.56% of total emissions. Electricity related emissions were modeled using the Turkish national electricity grid mix, which is still partially dependent on fossil fuel based energy sources. As a result, indirect emissions from electricity generation contribute measurably to the overall climate change impact, even when electricity consumption per functional unit remains relatively low. This finding is consistent with previous LCA studies conducted under Mediterranean conditions, where regional electricity mix characteristics significantly influence the carbon footprint of irrigated crop production [1, 33].

The combined contribution of irrigation water use and electricity consumption highlights the importance of energy efficiency in irrigation practices. Although irrigation related emissions did not represent the dominant hotspot in the present study, their indirect contribution through energy demand has been emphasized in recent Mediterranean assessments [2, 30].

From a mitigation perspective, potential options for the Silifke region include the adoption of energy efficient pumping systems, optimization of irrigation scheduling, and the integration of renewable energy sources such as solar powered irrigation systems. Such measures have been identified as effective strategies for reducing irrigation related greenhouse gas emissions in Mediterranean agriculture, particularly under water scarce and energy constrained conditions [11, 12].

##### 3.2.2.3 Agricultural Machinery and Diesel Use

The use of agricultural machinery and the associated diesel fuel consumption represents a notable contributor to the carbon footprint of open‐field strawberry production in the Silifke district. Mechanized operations considered within the system boundaries include land preparation, soil tillage, bed formation, and other field activities performed using tractors and related equipment.

According to the LCA results, the use of agricultural machinery generated 0.01597 kg CO_2_‐eq, accounting for 6.38% of the total carbon footprint (Table 4). These emissions primarily arise from the direct combustion of diesel fuel during field operations, as well as from indirect emissions related to fuel production and distribution. Similar contributions of mechanization related emissions have been widely reported for Mediterranean agricultural systems, where diesel consumption during soil preparation and field management constitutes a significant source of greenhouse gas emissions [30, 31].

In open‐field cropping systems, the magnitude of machinery related emissions depends strongly on the intensity of field operations, machinery efficiency, and fuel consumption rates. Previous studies conducted under Mediterranean conditions consistently identify soil tillage and land preparation as energy intensive processes that contribute substantially to climate change impacts [1]. The findings of the present study indicate that agricultural machinery use constitutes a secondary hotspot, following fertilizer use and irrigation related energy demand, which is consistent with the hotspot hierarchy reported for a wide range of Mediterranean crop types [33].

Comparable patterns have been observed across different Mediterranean cropping systems. For example, Garofalo et al. [30] reported that fuel combustion associated with tillage and field operations was one of the main contributors to greenhouse gas emissions in durum wheat production, while Roussis et al. [31] identified mechanized field operations as a dominant energy related hotspot in open‐field vegetable systems. The consistency of these findings across crop types highlights the structural role of diesel based mechanization in shaping the carbon footprint of Mediterranean agriculture.

From a mitigation perspective, emissions associated with agricultural machinery use could be reduced through the adoption of fuel efficient equipment, optimization of field operations, and the implementation of reduced or conservation tillage practices. Such strategies have been identified as effective options for lowering diesel consumption and associated greenhouse gas emissions in Mediterranean agricultural systems without compromising crop productivity [1, 11].

##### 3.2.2.4 Minor Inputs and Auxiliary Processes

Minor inputs and auxiliary processes contributed only marginally to the overall carbon footprint of open‐field strawberry production in the Silifke district. These inputs include fungicides, rooting fertilizers, and transportation of labor during the harvest period.

As shown in Table 4, fungicide application resulted in 0.00049 kg CO_2_‐eq, corresponding to 0.20% of total emissions, while rooting fertilizers contributed 0.00116 kg CO_2_‐eq (0.46%). Emissions associated with labor transportation during harvest were estimated at 0.00028 kg CO_2_‐eq, accounting for 0.12% of the total carbon footprint. These results clearly indicate that auxiliary inputs and logistical activities play a negligible role in shaping the overall greenhouse gas emissions of the production system when compared to fertilizers, irrigation related energy use, and machinery operations.

Similar findings have been reported in LCA studies of open‐field fruit and vegetable production systems under Mediterranean conditions, where auxiliary inputs such as plant protection products and labor transportation consistently contribute only a minor share to total climate change impacts [2, 19]. These studies emphasize that, although such inputs are necessary for crop protection and operational continuity, their relative influence on greenhouse gas emissions remains limited.

The inclusion of minor inputs and auxiliary processes in the life cycle inventory enhances the completeness and transparency of the assessment, allowing for a more comprehensive representation of the production system. However, given their limited contribution, mitigation efforts targeting these inputs are expected to yield only marginal reductions in the overall carbon footprint. Instead, the results support the prioritization of mitigation strategies focusing on dominant emission sources, in line with recommendations from recent Mediterranean agricultural sustainability assessments [11, 12].

### 3.3 Scenario‐Based Mitigation Analysis

Based on the scenarios defined in Section 2.4.1, the mitigation potential of key emission sources was evaluated (Table 5). Scenario 1 represents the baseline system reflecting current farming practices in the study area. Scenario 2 assesses the effect of reduced fertilizer application by decreasing NPK inputs by 20% relative to baseline conditions. Scenario 3 evaluates energy‐related mitigation by replacing grid electricity with renewable energy sources (solar‐powered irrigation), thereby eliminating electricity‐related emissions at the operational level. Scenario 4 combines both strategies, integrating fertilizer reduction and renewable energy use to represent a maximum mitigation scenario. These scenarios were designed to identify the relative contribution of different mitigation pathways and to quantify their effects on the overall carbon footprint.

**TABLE 5:** Scenario‐based carbon footprint results and mitigation potential of key inputs.

Input	Scenario 1 (kg CO_2_‐eq kg^−^^1^)	Scenario 2 (Fert)(kg CO_2_‐eq kg^−^^1^)	Scenario 3 (Solar)(kg CO_2_‐eq kg^−^^1^)	Scenario 4 (Combined)(kg CO_2_‐eq kg^−^^1^)
NPK fertilizers	0.12322	0.0986	0.12322	0.0986
Electricity consumption	0.0014	0.0014	0	0
Other inputs (total)	0.1259	0.1259	0.1259	0.1259
**Total CF**	**0.25052**	**0.2259**	**0.24912**	**0.2245**
**Change (%)**	**0**	**−9.83**	**−0.56**	**−10.39**

^*^Other inputs = Total CF—NPK—Electricity consumption.

The scenario analysis results indicate that fertilizer use is the dominant contributor to the total carbon footprint and offers the highest mitigation potential. A 20% reduction in NPK fertilizer application (Scenario 2) resulted in a decrease of 9.83% in total carbon footprint, highlighting the effectiveness of improved nutrient management practices. In contrast, the substitution of grid electricity with solar energy (Scenario 3) led to a relatively minor reduction (0.56%), reflecting the low contribution of electricity consumption within the overall system. The combined scenario (Scenario 4) achieved the highest reduction (10.39%), indicating that integrating multiple mitigation strategies provides additional benefits, although the overall reduction is still primarily driven by fertilizer optimization. These findings demonstrate that emission reduction strategies in open‐field strawberry production should prioritize nutrient management, while energy‐related interventions play a secondary role under the current system configuration. This outcome is consistent with previous studies emphasizing fertilizer management as the primary leverage point for emission reduction in agricultural systems.

### 3.4 Sensitivity Analysis

#### 3.4.1 Input‐Based Sensitivity

In addition to the scenario analysis presented in Section 3.3, which evaluates the impact of realistic mitigation strategies, a sensitivity analysis was conducted to assess the robustness of the model results. While scenario analysis focuses on system‐level changes, sensitivity analysis quantifies the influence of variations in individual input parameters on the overall carbon footprint.

Based on the hotspot analysis, NPK fertilizer use and electricity consumption were identified as the most influential parameters. Each parameter was varied within a ±20% range to reflect realistic variability in agricultural practices. In addition, a −100% variation in electricity consumption was included in Table 6 for comparative purposes, representing full substitution with renewable energy as defined in Scenario 3. This value is not part of the sensitivity coefficient calculation but is presented to facilitate direct comparison between sensitivity and scenario results.

**TABLE 6:** Sensitivity analysis of key input parameters (NPK fertilizers and electricity consumption) using a deterministic one‐at‐a‐time (OAT) approach.

Parameter	Change	CF (kg CO_2_‐eq kg^−^^1^)	Change (%)
Baseline	—	0.25052	0
NPK fertilizers	−20%	0.22590	−9.837
NPK fertilizers	20%	0.27516	9.837
Electricity consumption	−20%	0.25024	−0.110
Electricity consumption	20%	0.25080	0.110
Electricity consumption	−100%	0.24912	−0.560

In this study, percentage changes were used to simplify the calculation, such that the sensitivity coefficient can also be expressed as the ratio of percentage change in carbon footprint to percentage change in the input parameter. Sensitivity coefficients were calculated for NPK fertilizer and electricity consumption using ±20% variations in the selected input parameters (Table 6).

The sensitivity analysis results demonstrate that the total carbon footprint is highly sensitive to variations in fertilizer input, whereas changes in electricity consumption have a negligible effect. A ±20% variation in NPK fertilizer application results in approximately ±9.8% change in total carbon footprint, confirming its dominant contribution to overall emissions. In contrast, a 20% increase in electricity consumption leads to only a marginal increase (0.11%) in carbon footprint. The −100% change in electricity consumption corresponds to Scenario 3 (renewable energy substitution) and is presented in Table 6 for comparison only. These findings indicate that fertilizer management is the primary driver of environmental impact in the studied system. To further quantify the influence of key parameters, normalized sensitivity coefficients were calculated based on the relative change in carbon footprint and input variation (Table 7).

**TABLE 7:** Normalized sensitivity coefficients and relative changes in carbon footprint for key input parameters.

Parameter	Variation	%ΔCF	Sensitivity coefficient (S)
NPK fertilizers	±20%	±9.837	**0.492**
Electricity consumption	±20%	±0.11	**0.0055**

Sensitivity coefficients (S) were calculated as the ratio of percentage change in carbon footprint to percentage change in input parameter. Results indicate that NPK fertilizer is the dominant factor (S ≈ 0.492), whereas electricity consumption has a negligible influence (S ≈ 0.0055).

#### 3.4.2 Yield Sensitivity Analysis

A yield‐based sensitivity analysis was conducted to evaluate the influence of crop productivity on the calculated carbon footprint. In this analysis, yield was varied by ±20% while total emissions were assumed constant, allowing the isolation of the effect of productivity on the carbon footprint independently from changes in input use (e.g., fertilizer application). The resulting changes were calculated based on the inverse relationship between yield and carbon footprint (CF = E/Y), as presented in Table 8. This approach enables the assessment of environmental performance sensitivity to variations in production efficiency.

**TABLE 8:** Yield sensitivity analysis based on functional unit (CF = E/Y).

Scenario	^*^Yield (relative)	CF (kg CO_2_‐eq kg^−^^1^)	Change (%)
Baseline	1.00	0.25052	0
−20% yield	0.80	0.31315	+25.00
+20% yield	1.20	0.20877	−16.70

* Yield values are expressed relative to the baseline (Y = 1.00). The carbon footprint was calculated using CF = E/Y, where total emissions (E) were assumed constant across all scenarios.

The results indicate that yield variation has a stronger influence on the carbon footprint than electricity consumption, highlighting the importance of production efficiency in environmental performance. However, its effect remains lower than that of NPK fertilizer use, which is identified as the dominant driver of emissions in the system (Table 6).

While a ±20% change in yield resulted in a −16.70% to +25.00% variation in carbon footprint due to the inverse relationship between yield and the functional unit, changes in fertilizer input directly affect emission intensity and therefore exert a more consistent and dominant influence. These findings confirm that, although improving yield can significantly enhance environmental performance, fertilizer management remains the most effective mitigation strategy in the studied production system.

### 3.5 Discussion

#### 3.5.1 Environmental Impact Profile and Hotspot Identification

The environmental impact profile of open‐field strawberry production is characterized by a pronounced concentration of greenhouse gas emissions within a limited number of production inputs. This pattern indicates that the overall climate change performance of the system is primarily governed by a small set of input‐intensive processes, rather than by a wide distribution of minor emission sources. Such a concentrated emission structure highlights the effectiveness of hotspot‐oriented mitigation strategies in agricultural LCA studies.

Within this structure, fertilizer use emerges as the dominant driver of emissions, reflecting the combined effects of energy‐intensive production processes and soil‐related nitrogen emissions. Irrigation‐related energy demand and diesel‐based agricultural machinery represent secondary contributors, indicating the importance of energy use in cultivation practices under Mediterranean open‐field conditions. In contrast, auxiliary inputs such as plant protection products and minor operational activities contribute only marginally to the overall carbon footprint, suggesting limited mitigation potential when targeted individually.

This hotspot configuration is consistent with findings reported in Mediterranean agricultural LCA studies, where fertilizer use, irrigation‐related energy consumption, and mechanized field operations are repeatedly identified as the main contributors to climate change impacts [1, 2, 4, 29–31]. The consistency of these patterns across different crop types and production systems supports the robustness of the present results and reinforces the relevance of targeted mitigation strategies focusing on key input categories.

#### 3.5.2 Comparison With Previous Studies

The carbon footprint obtained for open‐field strawberry production in the Silifke district (0.25052 kg CO_2_‐eq kg^−^^1^) falls within the lower range of values reported in previous LCA studies, particularly when compared to protected and soilless cultivation systems. Strawberry‐specific LCA studies conducted under Mediterranean conditions consistently report higher climate change impacts for protected systems due to infrastructure materials, energy use, and input intensity. For example, Ilari et al. [34] reported significantly higher global warming potential values for tunnel‐based and soilless systems, while Clark and Mousavi‐Avval [35] highlighted the contribution of structural materials such as aluminum and plastics to overall emissions. These findings confirm that infrastructure‐related processes represent a key differentiating factor between open‐field and protected production systems. Comparative studies across different production systems further support this pattern. Frakolaki et al. [27] demonstrated that open‐field cultivation systematically results in lower emissions than protected systems, while Galafton et al. [28] emphasized the dominant role of infrastructure‐related emissions in greenhouse‐based production. These results are fully consistent with the findings of the present study. Within open‐field systems, variability in reported carbon footprint values is largely associated with differences in yield levels and input intensity. Romero‐Gámez and Suárez‐Rey [36] reported higher emission intensities under conditions of low productivity and elevated fertilizer application rates, highlighting the sensitivity of results to management practices. In contrast, the relatively lower carbon footprint obtained in this study reflects a combination of favorable productivity and efficient input use relative to crop yield. In this context, optimization refers to achieving higher productivity per unit of input rather than reducing absolute input quantities. Regional studies conducted in Türkiye provide additional context. Baran et al. [32] and Yılmaz et al. [37] reported values of a similar order of magnitude, although differences in climatic conditions, methodological approaches, and input structures lead to some variability in results. These comparisons confirm that the findings of the present study are consistent with regional production conditions. Beyond strawberry‐specific systems, similar emission ranges have been reported for other irrigated Mediterranean crops. Studies by Roussis et al. [31] and Tomaz et al. [29] demonstrate that fertilizer use, irrigation‐related energy demand, and mechanized field operations consistently dominate greenhouse gas emissions across Mediterranean agricultural systems. This structural similarity reinforces the representativeness of the present results. Meta‐analytical evidence further supports this interpretation. Mandouri et al. [2] and Pergola et al. [5] emphasize that carbon footprint values for fruit and vegetable systems vary widely depending on system boundaries and regional conditions, but generally fall within comparable ranges for open‐field systems. Overall, the comparison with previous studies indicates that the carbon footprint of open‐field strawberry production in Silifke is consistent with values reported for input‐intensive Mediterranean cropping systems. The relatively lower emissions observed in this study are primarily associated with the absence of energy‐intensive infrastructure and the use of optimized management practices, rather than methodological differences. These findings support the robustness and external validity of the present LCA results and confirm their relevance for Mediterranean open‐field production systems.

#### 3.5.3 Implications for Sustainable Strawberry Production in Silifke

The findings of this study highlight the importance of targeted mitigation strategies for improving the environmental performance of open‐field strawberry production systems under Mediterranean conditions. Given the concentration of emissions within a limited number of key inputs, mitigation efforts should primarily focus on improving resource use efficiency rather than broad system‐wide interventions. Fertilizer use, particularly NPK fertilizers, represents the most critical leverage point for emission reduction. Improving nutrient use efficiency through soil analysis‐based fertilization, optimized application timing, and the avoidance of over‐fertilization can significantly reduce greenhouse gas emissions associated with nitrogen inputs while maintaining yield stability. These approaches are widely recognized as cost‐effective and climate‐smart strategies for Mediterranean agricultural systems [1, 11].

Irrigation‐related emissions further emphasize the role of energy efficiency in water management. Although drip irrigation systems are already widely adopted, indirect emissions associated with electricity use for water pumping remain relevant. The integration of energy‐efficient pumping systems and renewable energy sources, particularly solar‐powered irrigation technologies, offers a promising pathway for reducing these emissions under Mediterranean climatic conditions [12]. Emissions from agricultural machinery and diesel consumption indicate additional opportunities for optimization. Improved planning of field operations, enhanced machinery efficiency, and reduced tillage intensity could lower fuel consumption without negatively affecting productivity. Such measures are consistent with mitigation strategies proposed for Mediterranean open‐field cropping systems facing increasing energy and climate constraints [1]. Overall, the results suggest that improving input efficiency, particularly in fertilizer and energy use, is essential for enhancing the environmental sustainability of strawberry production systems. These targeted interventions can support the transition toward more resource‐efficient and climate‐resilient agricultural practices at both farm and regional scales.

## 4 Limitations and Future Perspectives

Despite providing valuable insights into the carbon footprint of open‐field strawberry production in the Silifke district, this study is subject to several limitations that should be considered when interpreting the results. First, the assessment focuses exclusively on climate change impacts, expressed as carbon footprint (kg CO_2_‐eq), while other environmental impact categories were not examined in detail within the scope of the present analysis. Although this targeted approach enables a clear and policy relevant evaluation of greenhouse gas emissions, it does not capture the full spectrum of potential environmental trade offs associated with strawberry production systems.

Second, the system boundary was defined as cradle‐to‐farm gate, and therefore post harvest processes such as storage, packaging, distribution, retail, and consumption were excluded from the analysis. While this approach is consistent with many agricultural LCA studies, the exclusion of downstream processes may lead to an underestimation of total life cycle impacts when strawberries are evaluated from a broader supply chain perspective.

In addition, inventory data were primarily based on field surveys and interviews with producers representing typical open‐field production practices in the Silifke region. Although this approach enhances the regional relevance and realism of the results, variations in farm management practices, input application rates, and yield levels across farms and production seasons may introduce uncertainty into the estimated emissions.

From a future research perspective, expanding the scope of the assessment to include additional environmental impact categories—such as water scarcity, eutrophication, and ecotoxicity—would allow for a more comprehensive evaluation of sustainability performance. Moreover, extending the system boundary beyond the farm gate and exploring alternative production scenarios, including optimized fertilizer management strategies, renewable energy based irrigation systems, and low input cultivation practices, would further support the development of climate‐resilient and environmentally sustainable strawberry production systems in the Silifke region.

The integration of scenario‐based mitigation analysis with sensitivity and yield‐based assessments provides a comprehensive understanding of both structural and parametric drivers of carbon footprint in agricultural production systems. While scenario analysis identifies feasible mitigation pathways under real‐world conditions, sensitivity analysis reveals the relative importance of individual inputs, and yield sensitivity highlights the critical role of productivity in determining environmental performance. By distinguishing between structural mitigation strategies (e.g., fertilizer reduction) and parametric drivers (e.g., yield variability), this combined analytical framework strengthens the robustness of the study and contributes to a deeper understanding of climate mitigation opportunities in Mediterranean horticulture, thereby supporting more informed decision‐making for climate‐smart agricultural systems.

## 5 Conclusions

This study evaluated the carbon footprint of open‐field strawberry production in the Silifke district (Mersin Province, Türkiye) using a process‐based life cycle assessment approach. The results show that the carbon footprint falls within the lower range reported for Mediterranean strawberry production systems, particularly in comparison with protected and soilless cultivation systems.

The analysis revealed a highly concentrated emission structure, where a limited number of inputs dominate the overall environmental impact. In particular, fertilizer use emerged as a key driver of greenhouse gas emissions, followed by irrigation‐related energy use and agricultural machinery. This pattern confirms that improving input efficiency represents the most effective strategy for emission reduction.

Scenario and sensitivity analyses consistently indicate that fertilizer management is the key leverage point for mitigation, while energy‐related interventions have a comparatively limited effect under current production conditions. In addition, yield performance plays a critical role in determining environmental outcomes, highlighting the importance of maintaining high productivity alongside efficient resource use.

The relatively low carbon footprint observed in this study is mainly associated with the absence of energy‐intensive infrastructure and the use of efficient input practices under open‐field conditions. These findings are consistent with previous LCA studies conducted in Mediterranean agricultural systems. The findings provide region‐specific insights into the environmental performance of open‐field strawberry production and highlight practical mitigation pathways based on fertilizer optimization, energy efficiency, and productivity improvement. These findings can support farmers, policymakers, and researchers in developing climate‐smart and resource‐efficient agricultural practices tailored to Mediterranean conditions.

## Funding

The authors have nothing to report.

## Conflicts of Interest

The authors declare no conflicts of interest.

## Data Availability

The data that support the findings of this study are available from the corresponding author upon reasonable request.
